# COMPARISON OF AGE AND BIOLOGICAL SEX MORTALITY TRENDS BETWEEN ADULTS WITH AND WITHOUT DOWN SYNDROME

**DOI:** 10.1093/geroni/igz038.1278

**Published:** 2019-11-08

**Authors:** Scott D Landes, Scott D Landes, James D Stevens, Margaret D Turk

**Affiliations:** 1 Syracuse University, Aging Studies Institute, Syracuse, New York, United States; 2 Syracuse University, Syracuse, New York, United States; 3 Upstate University Hospital, Syracuse, New York, United States

## Abstract

Age at death and cause of death comparisons between adults with and without Down syndrome reveal distinct mortality trends that can be utilized to inform preventive care efforts to reduce premature mortality in this population. We compare mean and median age at death, and standardized mortality odds ratios (SMORs) for 20 leading causes of death for 9,564 decedents with and 13,050,319 without Down syndrome in the U.S. between 2012 and 2016. Decedents with Down syndrome, on average, were substantially younger than those without Down syndrome, and were more likely to die from Alzheimer disease and dementia at younger ages. In addition, adults with Down syndrome also had higher risk of choking related deaths. Efforts to reduce premature mortality through public health and preventive care interventions for this population should be cognizant of these increased risk factors, as well as variation in age and biological sex mortality trends.

